# Performance of MIL-101(Cr)/Water Working Pair Adsorption Refrigeration System Based on a New Type of Adsorbent Filling Method

**DOI:** 10.3390/ma13010195

**Published:** 2020-01-02

**Authors:** Zhongbao Liu, Banghua Zhao, Longqian Zhu, Fengfei Lou, Jiawen Yan

**Affiliations:** 1 Department of Refrigeration and Cryogenic Engineering, College of Environmental and Energy Engineering, Beijing University of Technology, 100 Pingleyuan Road, Chaoyang, Beijing 100124, China; zhaobanghua@emails.bjut.edu.cn (B.Z.); loufengfei@emails.bjut.edu.cn (F.L.); yanjiawen@emails.bjut.edu.cn (J.Y.); 2 Fok Ying Tung Graduate School, The Hong Kong University of Science and Technology, Clear Water Bay, Kowloon, Hong Kong; lzhuac@connect.ust.hk

**Keywords:** metal–organic frameworks, MIL-101 (Cr)/water, refrigeration, adsorption performance test rig

## Abstract

MIL-101(Cr) and water were applied to adsorption refrigeration technology. MIL-101(Cr) was prepared by hydrothermal synthesis method and characterized by X-ray diffraction patterns (XRD), Fourier transform infrared spectroscopy (FTIR), N_2_ adsorption–desorption measurement at 77 K, thermal gravimetric analysis (TGA) and scanning electron microscope (SEM). The adsorption isotherms of water vapor on MIL-101(Cr) were investigated by using a gravimetric water sorption analyzer. This study established the basic adsorption cycle mathematical model and used MATLAB/Simulink for the simulation. The control variable method was used to simulate the effect on the cooling capacity and coefficient of performance (COP) when the desorption temperature changed. When the adsorption temperature was 35 °C, the evaporation temperatures were 15 °C and 20 °C, and the amount of water vapor equilibrium adsorption on MIL-101 (Cr), Cooling power per unit adsorbent mass (SCP), and COP were measured by using the adsorption performance test rig on the basis of a new type of powder adsorbent filling method.

## 1. Introduction

At present, considerable research focuses on thermally driven adsorption chillers (TDCs) or adsorption heat pumps (AHPs) because they fully exploit environmentally friendly refrigerants (e.g., water and ethanol) and low-grade thermal energy (e.g., solar energy and industrial waste heat) [1,2,3,4]. The traditional work on the application of zeolites-water and silica gel-water in TDCs and AHPs was studied. It was found that the performance coefficient (COP) was low and the cycle stability was poor [5,6,7]. Therefore, this research topic remains crucial for developing new solid adsorbent materials with excellent adsorption performance for AHPs [8]. Metal–organic frameworks (MOFs) are a novel micro-mesoporous solid adsorbent material with great potential in TDCs or AHPs given their high specific surface area (4000 m^2^·g^−1^) and porosity [9]. Küsgens [10] studied the adsorption isotherm of water vapor and water stability for four kinds of MOFs materials. MIL-101 (Cr) had a high adsorption capacity of water vapor and improved water stability and had the most potential in water adsorption applications. The adsorption effect of MIL-101 (Cr) on different adsorbates is also different. MIL-101 (Cr) has the largest adsorption capacity for ethanol (1.2 g·g^−1^), and the MIL-101 (Cr)/ethanol working pair has a remarkable stability [11]. Modification of MIL-101 (Cr) or compounding with different materials will improve the water vapor adsorption capacity of the original MIL-101 (Cr) [12,13,14]. Although many reports about the adsorption characteristics of MOF materials are available [15,16,17,18,19], researchers have only used a gravimetric water sorption analyzer to investigate the adsorption performance, and the application of MIL-101 (Cr)/water to the actual refrigeration test bench to test the adsorption performance is scarce [20,21,22]. For the actual adsorption performance test, especially in a vacuum system, powder adsorbent is typically pressed into a block or granule. Then, the adsorbent’s performance, which will not fully reflect the adsorption properties of the powder adsorbents, is investigated.

Given these disadvantages, in the present study, MIL-101 (Cr) powder adsorbent was synthesized by hydrothermal synthesis and characterized by X-ray diffraction patterns (XRD), Fourier transform infrared spectroscopy (FTIR), N_2_ adsorption–desorption measurement at 77 K, thermal gravimetric analysis (TGA), and scanning electron microscope (SEM). The water vapor adsorption isotherms were obtained by using a gravimetric water sorption analyzer. Furthermore, MIL-101 (Cr) and water used as a working pair were applied to AHPs. The adsorption performance test rig was built on the basis of the liquid level method, and a new type of powder adsorbent filling method was used to experimentally study the adsorption performance of MIL-101 (Cr) powder/water working pair.

## 2. Experiment

### 2.1. Reagents and Materials

1,4-benzenedicarboxylic acid (H2BDC, ≥99.0% purity; Beijing Sinopharm Chemical Reagent), Chromium nitrate nonahydrate (Cr(NO_3_)_3_·9H_2_O, ≥99% purity; Braunwell Technology Co., Ltd., Beijing, China), hydrofluoric acid (HF, ≥40% purity; Tianjin Kermel Chemical Reagent Co., Ltd., Beijing, China), N,N-Dimethylformamide (DMF, ≥99% purity; Braunwell Technology Co., Ltd., Beijing, China), ammonium fluoride (NH_4_F, ≥96% purity; Beijing Sinopharm Chemical Reagent, Beijing, China), ethanol (≥99.7% purity; Beijing Sinopharm Chemical Reagent, Beijing, Chinaand distilled water (homemade in the laboratory of Beijing University of Technology, Beijing, China).

### 2.2. Material Synthesis and Characterization

Considerable MIL-101 (Cr) was hydrothermally synthesized for adsorption refrigeration, following the steps reported by Teo [23]. The XRD pattern was obtained by scanning MIL-101 (Cr) with an XD-6 X-ray diffractometer (Beijing Puri General Instruments Co., Ltd., Beijing, China) with a Cu-Kα radiation source (λ = 0.15432 nm) which has a scanning speed of 2°/min and a scanning range 2θ between 2° and 20°. N2 adsorption–desorption isotherm was measured by JW-BK300 full-automatic specific surface area analyzer (Beijing Jingwei Gaobo Science and Technology Co., Ltd., Beijing, China) at 77 K temperature, the specific surface area was calculated in the range of 0.05–0.3 relative pressure. The pore size distribution curve was obtained from the adsorption isotherm by Barrett–Joyner–Halenda method. The FTIR spectrum in the wavenumber range of 400–4000 cm^−1^ was conducted with IR Prestige-21 using KBr wafer technique. HS-TGA-10 thermogravimetric analyzer (Shanghai Hesheng Technology Co., Ltd., Shanghai, China) was used for TGA. The test conditions were set as follows: nitrogen flow at 30 mL/min, temperature range was 30–900 °C, and the heating rate was set as 5 °C/min. The SEM that operates at 25 kV and utilizes JEOLJEM-220 was used to investigate surface morphology.

### 2.3. Measurement of Water Vapor Adsorption Isotherms

The water vapor adsorption isotherm was measured by a 3H-2000PW gravimetric water adsorption analyzer (Beishide Instrument Technology (Beijing) Co., Ltd., Beijing, China), which is mainly composed of a vacuum control system, a micro-sky platform, a constant temperature tank, a degassing furnace, and other devices. The measurable adsorption temperature range is from room temperature to 50 °C, the degassing temperature range is from room temperature to 400 °C, the balance accuracy can reach ± 1.0 μg, and the system vacuum is controlled below 0.01 Torr.

Test steps for the water vapor adsorption isotherm: a sample of about 50.0000 mg was weighed and dried in a vacuum for 12 h at 423 K, the water vapor adsorption temperature, relative humidity range, and water vapor saturation pressure at the corresponding temperature were set, and the water vapor adsorption analyzer was turned on for testing. 

### 2.4. Experimental Rig of Single-Bed Adsorption System with New Type Adsorbent Filling Method

**Figure 1:**
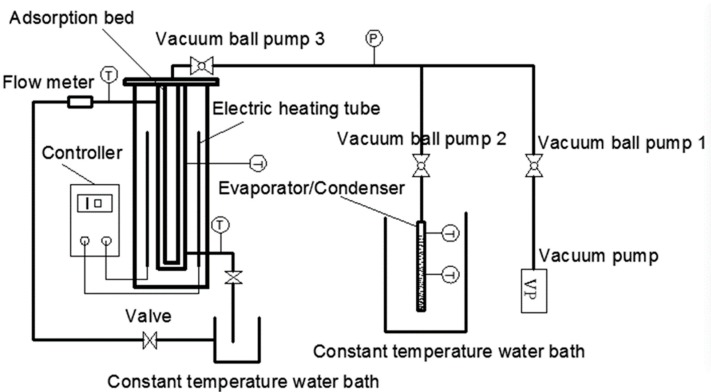
Schematic of the adsorption performance test.

**Figure 2:**
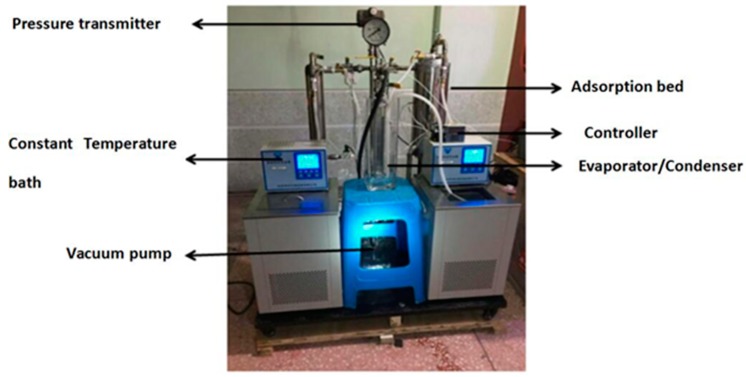
Test rig of the adsorption performance test.

A single-bed adsorption system was mainly comprised of heating/cooling unit, adsorption bed, evaporator/condenser, vacuum system, and data acquisition system (Figure 1 and Figure 2). The heating unit mainly consisted of a furnace made of two electric heating tubes and a temperature controller. The cooling unit mainly used a constant temperature water bath to cool the adsorption bed, and the temperature range and accuracy of the constant temperature water bath were −5–100 °C and 0.1 °C, respectively. The adsorption bed was mainly made of a stainless steel tube, and the outside diameter, inside meter, and height were 58, 52, and 300 mm, correspondingly. The evaporator/condenser was mainly made of quartz glass with a scale, and the outside diameter, inside meter, and height were 28, 25, and 300 mm, respectively. The air in the system and the water vapor of MIL-101 (Cr) were extracted using a vacuum pump. The pumping rate and vacuum degree were 3 L/s and 0.1 Pa, correspondingly. The power of heater for adsorption was 125 W. The filling mass of adsorbent was 100 g. The data acquisition system was mainly composed of thermal resistance, pressure transmitter (the main technical parameters are listed in Table 1), and data acquisition instrument.

**Figure 3:**
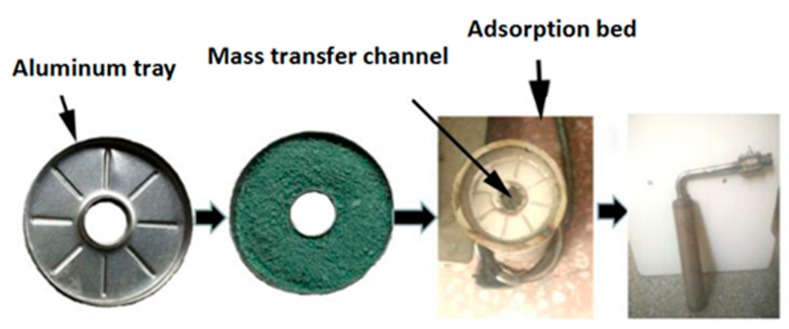
Filling method of an adsorbent.

**Figure 4:**
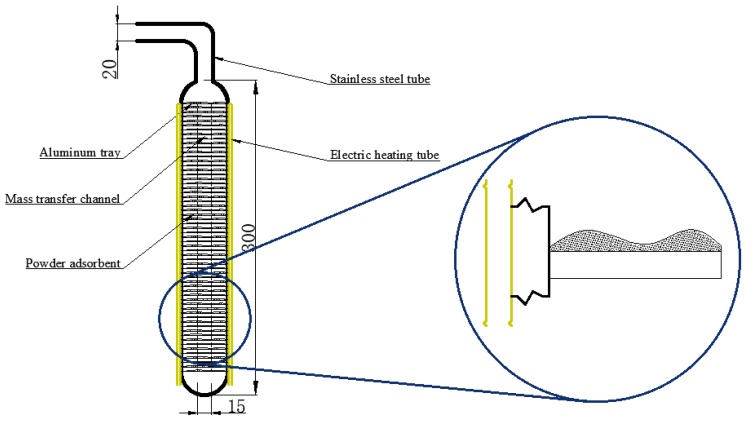
Vertical section of the filled adsorption bed.

**Figure 5:**
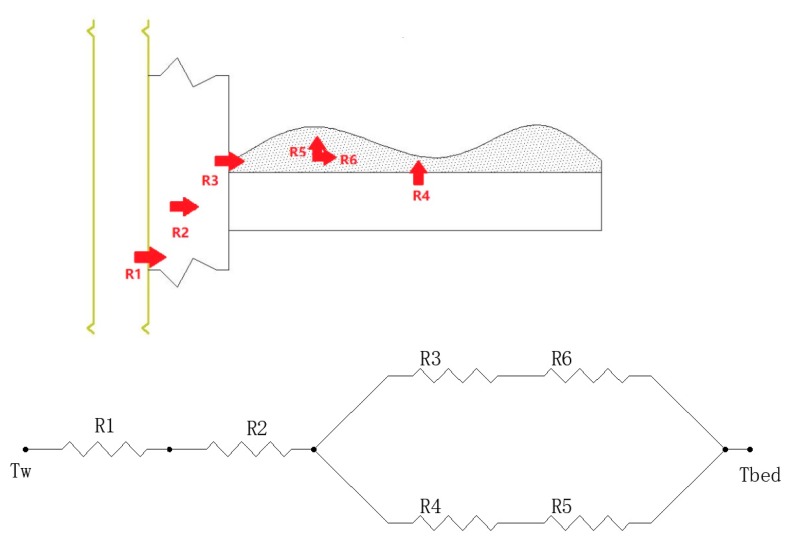
Schematic diagram of heat transfer process.

This study proposes a new filling method that can be used to fill the powder adsorbent. The way of filling is illustrated in Figure 3. In the filling process, the powder MIL-101 (Cr) adsorbent was initially installed in the homemade aluminum tray, and then a tray that contained the powder MIL-101 (Cr) adsorbent was placed in an adsorption bed made of stainless steel. Finally, the adsorption bed was welded with the pipe. The vertical section of the filled adsorption bed is shown in Figure 4. There were six thermal resistances in the heat transfer process based on this new filling method (ignoring the thermal resistance of the aluminum tray to the surrounding air): (1) electric heating tube heat transfer to stainless steel tube thermal resistance R1; (2) Stainless steel tube wall thermal resistance R2; (3) The thermal resistance of the adsorbent powder in contact with the outer surface of the tube and the tray were R3 and R4, respectively; (4) The radial and axial guide thermal resistance of the adsorbent powder were R5 and R6, respectively. Figure 5 shows the heat transfer process. This finned heat transfer structure increases the contact area between the adsorbent and the bed body and reduces the contact thermal resistance R4.

A schematic of the adsorption performance test is depicted in Figure 1, and the testing steps are presented as follows.

(1) The adsorption bed was initially heated by the temperature controller, and the adsorption bed temperature reached 120 °C after approximately 10 min. Then, the vacuum pump and vacuum ball valves one and three were opened to heat the adsorption bed. When the pressure of the adsorption bed was close to that of the vacuum, vacuum ball valve three and the electric heating tube were turned off. Finally, the vacuum ball valve two was opened and, while closing the valve and the vacuum pump, the excess air was extracted from the evaporator/condenser until the pressure was the pressure at the evaporation temperature/condensation temperature.
(2) The thermostat tank was set as the corresponding adsorption temperature, the circulating water valve on the left of Figure 1 was opened, then the vacuum ball valve two and three were opened, the adsorption bed and evaporator/condenser could form a closed system. Next, a refrigerant (water) began to evaporate rapidly. Moreover, MIL-101 (Cr) began to absorb the water vapor, the heat released in the adsorption process was taken away by the circulating cooling water. The liquid level of the evaporator at the beginning and end was recorded.
(3) Vacuum ball valves two and three were closed after completing Step (2). Then, the adsorption bed temperature was set to desorption temperature (100 °C) using the temperature controller. The temperature of the constant temperature water bath was set to condensing temperature (30 °C). After 30 min, vacuum ball valves two and three were opened, and the process of desorption–condensation began.

## 3. Mathematical Model of the Single-Bed Adsorption System

### 3.1. Basic Assumptions of the Theoretical Model

In the adsorption refrigeration cycle, the energy transfer process is extremely complicated in the stage of adsorption–evaporation and desorption–condensation. To simplify the complicated calculation of mathematical modeling, the lumped parameter method is adopted for the system simulation, and the following assumptions are formed: (1) The temperature and steam pressure inside the adsorption bed are uniform; (2) water is uniformly adsorbed by MIL-101 (Cr) and is in a liquid state inside MIL-101 (Cr); (3) the pressure difference between the adsorption bed and evaporator/condenser is neglected; (4) the heat exchange between the refrigerant passage and the external environment that occurs between the adsorption bed and the evaporator/condenser is neglected; (5) the adsorption bed is completely insulated from the evaporator/condenser; (6) the heat lost to the outside environment is neglected; and (7) the specific heat capacity of MIL-101 (Cr), the specific heat capacity of adsorption bed, and the specific heat capacity of refrigerant are all constant.

### 3.2. Mathematical Model of the Basic Cycle 

The heat absorbed during the desorption process [24] is

(1) $$Q_{g}=\int_{T_{g1}}^{T_{g2}}C_{a}\cdot M_{a}\cdot dT+\int_{T_{g1}}^{T_{g2}}C_{lc}\cdot M_{a}\cdot x\cdot dT+\int_{T_{g1}}^{T_{g2}}C_{m}\cdot M_{m}\cdot dT+\int_{T_{g1}}^{T_{g2}}M_{a}\cdot h_{d}\cdot dT$$

The heat taken away during cooling of adsorption bed is

(2) $$Q_{c}=\int_{T_{a1}}^{T_{g2}}C_{a}\cdot M_{a}\cdot dT+\int_{T_{a1}}^{T_{g2}}C_{lc}\cdot M_{a}\cdot x_{d\mathrm{il}}\cdot dT+\int_{T_{a1}}^{T_{g2}}C_{m}\cdot M_{m}\cdot dT$$

The cooling capacity is

(3) $$Q_{ref}=M_{a}\cdot L\cdot △x$$

The sensible heat released by liquid refrigerant from *T_c_* down to evaporation temperature *T_e_* is

(4) $$Q_{\mathrm{eva}}=M_{a}\cdot C_{lc}\cdot △x$$

The circulating adsorption capacity is

(5) $$△x=x_{conc}-x_{dil}$$

The system COP is

(6) $$COP=\frac{Q_{ref}-Q_{eva}}{Q_{h}+Q_{g}}$$

For physical adsorption, the Dubinin–Astakhov equation describes the relationship between adsorption capacity and adsorption conditions (Equation (7)) [25]. Since the adsorption amount at the adsorption temperature and the initial desorption temperature are equal, the initial desorption temperature can be obtained by substituting the two states into the D–A equation respectively (Equation (8)). Similarly, since the adsorption amount at the desorption temperature and the initial adsorption temperature is the same, the initial adsorption temperature can be obtained by substituting the two states into the D–A equation respectively (Equation (9)). Desorption heat h_d_ is also a function of temperature (Equation (10)).

(7) $$x(T,T_{sat})=x_{0}\exp[-K{\left(T/T_{sat}-1\right)}^{n}]$$

(8) $$T_{g1}=\frac{T_{c}T_{a2}}{T_{e}}$$

(9) $$T_{a1}=\frac{T_{c}T_{g2}}{T_{e}}$$

(10) $$h_{d}(T,T_{c})=RA\frac{T}{T_{c}}$$

The parameters used in the simulation are summarized in Table 2.

## 4. Results and Discussion 

### 4.1. XRD and FTIR Analysis

**Figure 6:**
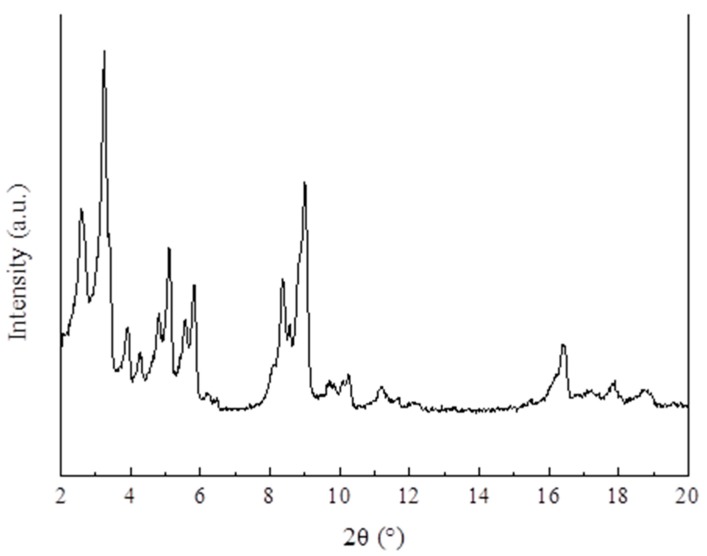
XRD patterns of MIL-101 (Cr).

Figure 6 demonstrates the XRD pattern of MIL-101 (Cr). The main diffraction peaks in the XRD pattern of MIL-101 (Cr) were found at 2θ = 3.24°, 5.82°, 8.36°, 9°, 10.24°, and 16.4°; the characteristic diffraction peaks and positions of the synthesized MIL-101 (Cr) are consistent with the standard pattern.

**Figure 7:**
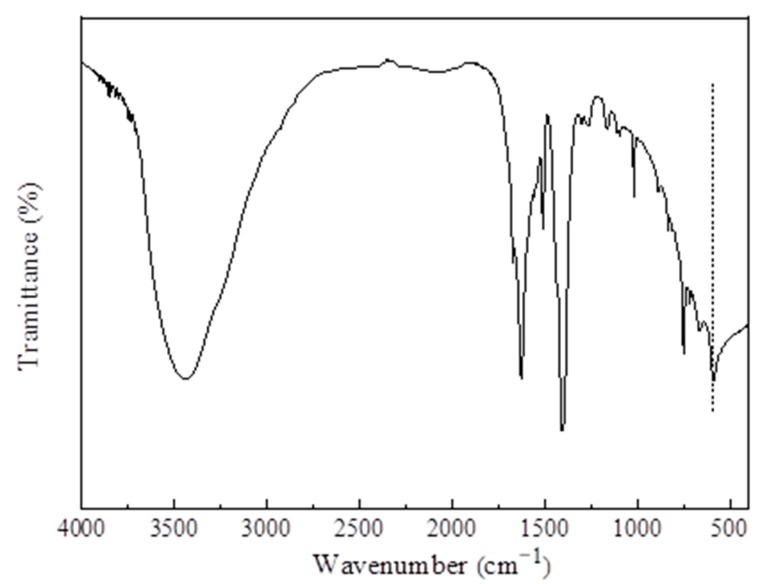
FTIR spectra of MIL-101 (Cr).

Figure 7 exhibits the FTIR spectrum of MIL-101 (Cr). As shown in the figure, the carboxyl antisymmetric and symmetric vibration peaks of BDC in MIL-101 (Cr) were located at 1510 and 1400 cm^−1^ respectively, indicating that the sample contained the organic ligand terephthalic acid. The wide peak at 3442 cm^−1^ may be due to the presence of water molecules or acid hydroxyl groups in the synthesized MIL-101 (Cr); The Cr–O vibration peak appeared at 585 cm^−1^, which further indicates the successful synthesis of MIL-101 (Cr). 

### 4.2. N_2_ Adsorption–Desorption Isotherms and Pore Size Analysis

**Figure 8:**
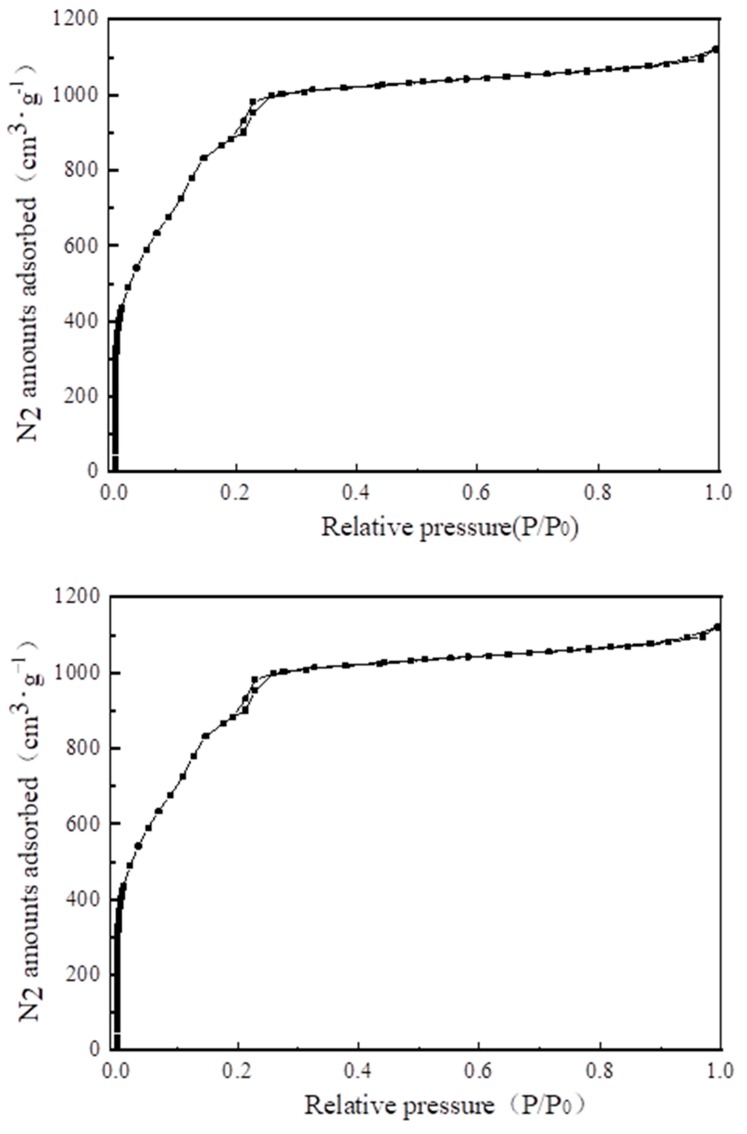
N_2_ adsorption–desorption isotherms of MIL-101 (Cr).

Figure 8 shows the N_2_ adsorption and desorption isotherm. In the range of relative pressure 0–0.1, N_2_ adsorption increased sharply, indicating that the synthesized MIL-101 (Cr) contained a large number of microporous structures. When the relative pressure was 0.2, a second adsorption occurred, indicating that MIL-101 (Cr) contained two different porous cage structures.

**Figure 9:**
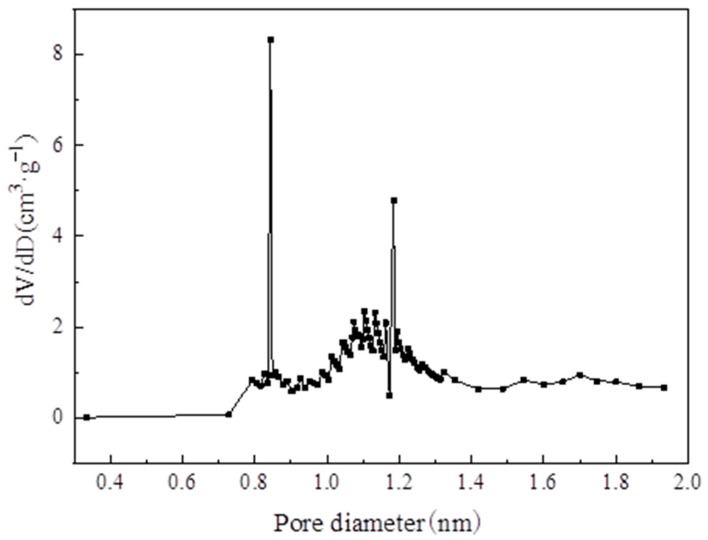
Pore diameter distribution of micropore on MIL-101 (Cr).

**Figure 10:**
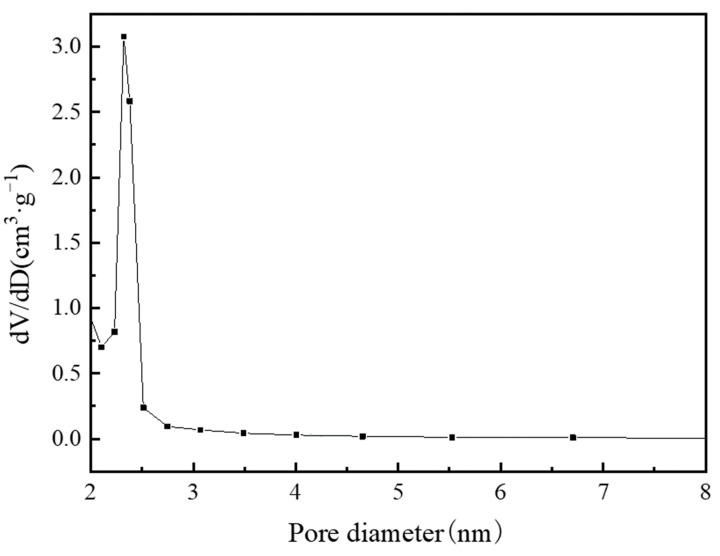
Pore diameter distribution of mesoporous on MIL-101 (Cr).

Table 3 lists the structural properties of MIL-101(Cr). The calculated BET specific surface area and pore volume of MIL-101 (Cr) were 3054 m^2^·g^−1^ and 1.734 cm^3^·g^−1^, respectively; these values are close to those of Li’s [22] investigation. Figure 9 and Figure 10 show the pore size distribution curve of MIL-101(Cr), and the pore size of MIL-101 (Cr) was mainly distributed at 0.85, 1.18, and 2.32 nm.

### 4.3. TGA and SEM Analysis

**Figure 11:**
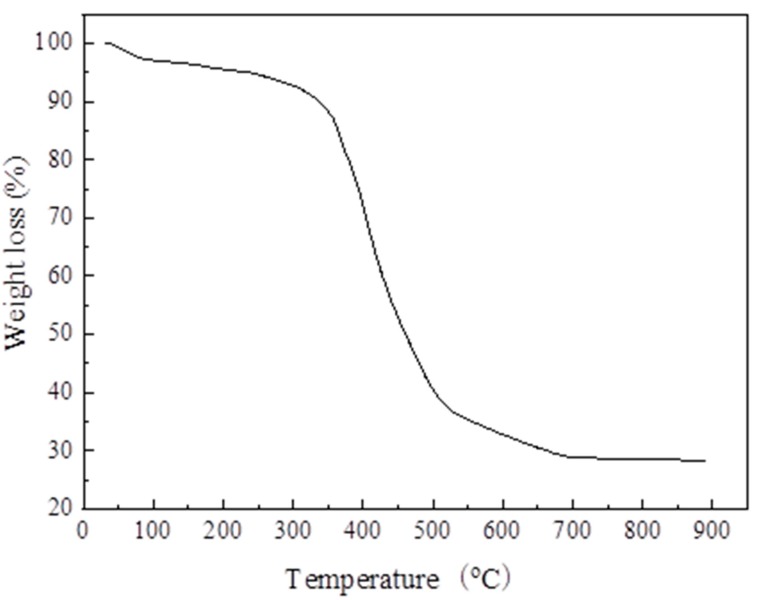
Thermo-gravimetric curve of MIL-101 (Cr).

Figure 11 shows the thermo-gravimetric curve of the sample. The weight loss of MIL-101 (Cr) can be divided into two parts. First, the weight loss rate was about 7% when the temperature was from 30 to 240 °C, mainly due to the loss of guest water molecules; Second, when the temperature was from 270 to 570 °C, the weight loss rate was about 58%, which was mainly due to the elimination of the OH/F functional groups in the skeleton, which led to the decomposition of the skeleton.

The SEM of MIL-101 (Cr) shows that MIL-101 (Cr) had an octahedral configuration with a relatively uniform crystal growth and a smooth surface.

### 4.4. Water Vapor Adsorption Isotherms

**Figure 12:**
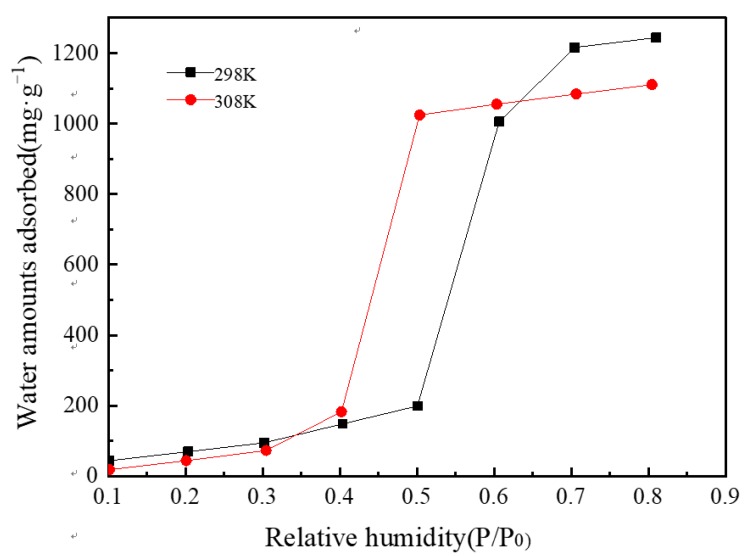
Water vapor adsorption isotherms of MIL-101 (Cr).

Figure 12 presents the water vapor adsorption isotherm diagram of MIL-101 (Cr) at 298 and 308 K. The adsorption isotherms were S-shaped, and the entire adsorption process could be divided into three steps. Using the adsorption isotherm at 308 K as an example, first, in the range of relative humidity of 0.1–0.3, the amount of water vapor adsorption increased linearly with relative humidity. This process is due to the connection of water molecules to the hydrophilic center of MIL-101 (Cr). Second, in the range of relative humidity of 0.3–0.5, the amount of water vapor adsorption increased sharply because water molecules were adsorbed into the mesopores of the MIL-101 (Cr) material. Third, in the range of relative humidity of 0.5–0.8, the amount of water vapor adsorption increased slowly because water molecules entered the gap of MIL-101 (Cr) powder. In addition, Figure 12 illustrates that the maximum water vapor adsorption capacity of MIL-101 (Cr) is approximately 1.2 and 1.1 g·g^−1^ at 298 and 308 K, correspondingly.

### 4.5. Analysis of the Simulation Results

**Figure 13:**
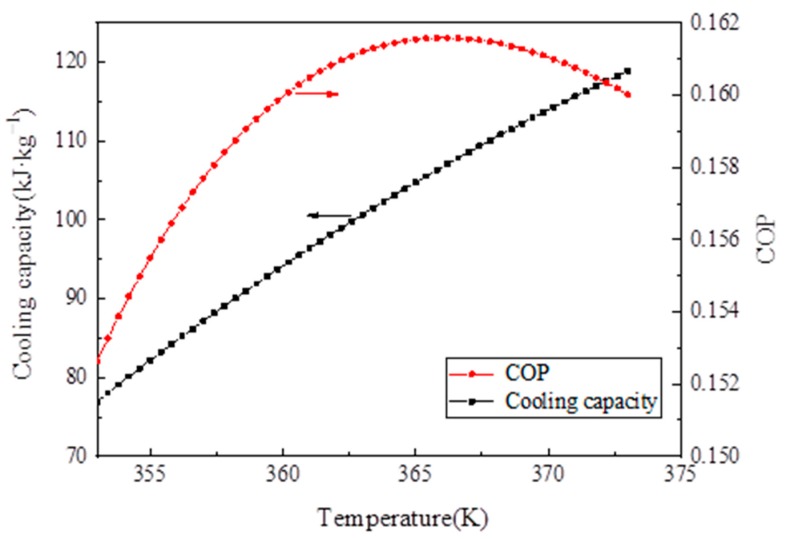
Influence of desorption temperature on the cooling capacity and coefficient of performance (COP) analysis of the experimental results.

Figure 13 depicts the effect of desorption temperature on cooling capacity and COP. As the desorption temperature increased, the cooling capacity increased. Initially, COP increased with desorption temperature. When desorption temperature reached 367 K, the COP of the system reached a maximum of 0.161 and then decreased because the desorption amount of the refrigerant remained unchanged when the temperature of 367 K was reached, but the sensible heat of the adsorption bed increased, thereby decreasing the system COP.

### 4.6. Analysis of the Experimental Results

#### 4.6.1. Changes in Adsorption Bed Temperature and System Pressure During Evacuation 

**Figure 14:**
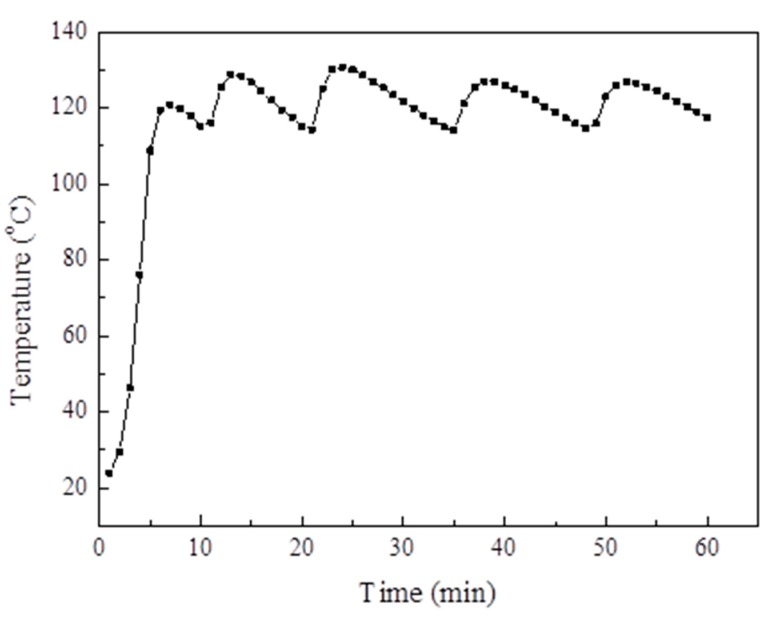
The adsorption bed temperature changes during the extraction process.

Figure 14 displays the changes in the adsorption bed temperature and system pressure during the evacuation, respectively. The adsorption bed temperature was heated for 1 h and increased from room temperature to 120 °C and then fluctuated at 120 °C. When the adsorption bed temperature reached 120 °C, the vacuum pump was opened. After 4 min, the pressure of the system dropped to 0.1 kPa and then maintained for 8 min, thereby indicating that the pressure of the system is close to that of the vacuum.

#### 4.6.2. Reliability Results of the Adsorption System

**Figure 15:**
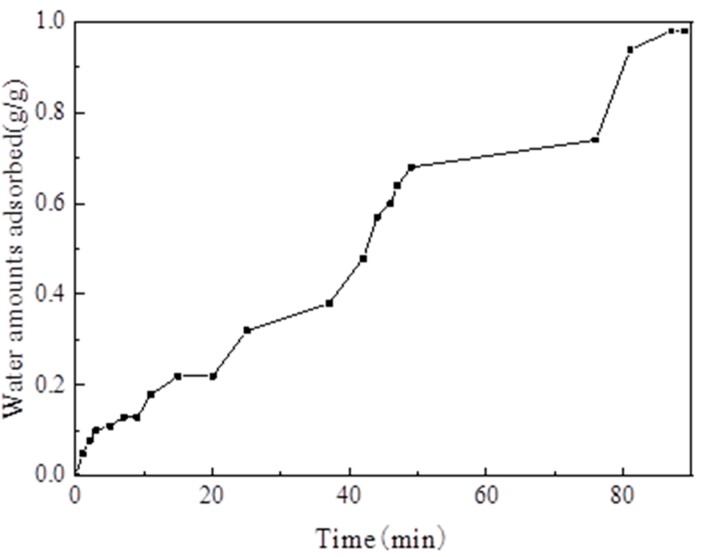
Adsorption uptake curve of water vapor versus time.

The reliability test of the adsorption system mainly aimed to compare the amount of water vapor adsorption measured by the test rig. with the data measured using the gravimetric water sorption analyzer to verify the reliability of the test rig. The test rig shows that the temperature of the evaporator and the adsorption bed was set to 35 °C, and the water vapor adsorption uptake curve versus time is presented in Figure 15. When the adsorption temperature was 35 °C, the amount of water vapor equilibrium adsorption on MIL-101 (Cr) was 0.98 g·g^−1^. When the adsorption temperature was 35 °C, the amount of water vapor equilibrium adsorption on MIL-101(Cr) measured using the gravimetric water sorption analyzer was 1.1 g·g^−1^. The relative error between the two methods was only 10.9%. This result is due to the readings and the backflow of water vapor into the evaporator by gravity during evaporation.

#### 4.6.3. System COP and SCP

**Figure 16:**
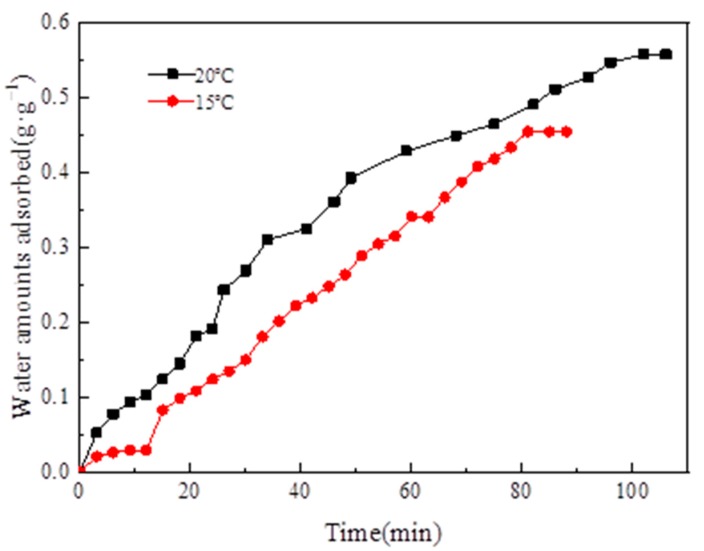
Adsorption uptake curve of water vapor versus time.

When the adsorption temperature was 35 °C, the adsorption–evaporation process was completed at the evaporation temperatures of 15 °C and 20 °C, correspondingly. The water vapor adsorption uptake curve versus time is illustrated in Figure 16.

Figure 16 depicts that, when the evaporation temperature was 15 °C, the amount of water vapor equilibrium adsorption was 0.45 g·g^−1^. When the evaporation temperature was 20 °C, the amount of water vapor equilibrium adsorption was 0.55 g·g^−1^. When the evaporation temperature was 20 °C, the amount of water vapor equilibrium adsorption was higher than the amount of water vapor equilibrium adsorption at 15 °C because evaporation temperature increased with evaporation pressure. The amount of water vapor equilibrium adsorption on MIL-101 (Cr) increased because the pressure difference between the evaporator and the adsorption bed increased.

The adsorption–desorption cycles for system COP and SCP were conducted at the adsorption temperature of 35 °C, the desorption temperature of 100 °C, the condensation temperature of 30 °C, and the evaporation temperatures of 15 °C and 20 °C, respectively (Table 4). Table 4 displays that, when the evaporator temperatures were 15 °C and 20 °C, the COPs are 0.112 and 0.144, and the SCPs were 42.7 and 43.4 W·kg^−1^, correspondingly.

## 5. Conclusions

(1) The XRD (2θ = 3.24°, 5.82°, 8.36°, 9°, 10.24°, and 16.4°) and FTIR spectroscopy results showed that the MIL-101 (Cr) material was successfully synthesized.
(2) The TGA results showed that the synthesized MIL-101(Cr) could be stabilized to 270 °C.
(3) The results of the water vapor adsorption test showed that the maximum water vapor adsorption capacities of MIL-101 (Cr) were approximately 1.2 and 1.1 g·g^−1^ at 298 and 308 K, respectively.
(4) The simulation results showed that, when the desorption temperature reached 367 K, the system COP reached a maximum value of 0.161 and then decreased with the increase in desorption temperature, whereas the cooling capacity increased with the desorption temperature.
(5) When the adsorption temperature was 35 °C, the evaporation temperatures were 15 °C and 20 °C, and the amounts of the water vapor equilibrium adsorption of MIL-101 (Cr) were 0.45 and 0.55 g·g^−1^. Moreover, the SCPs were 42.7 and 43.4 W·kg^−1^, and the system COPs were 0.112 and 0.144.

## Figures and Tables

**Table 1:** Main technical parameters of the pressure transmitter.

Content	Value
power Supply (V)	24
operating temperature range (°C)	−30–80
signal (mA)	4–20
gauge pressure range (kPa)	−100–1600
accuracy (kPa)	0.1

**Table 2:** Parameters used in the simulation.

Symbol	Value	Unit
*M_a_*	0.1	Kg
*M_m_*	2	Kg
*C_lc_*	4.18	kJ·kg^−1^·K^−1^
*C_m_*	0.5	kJ·kg^−1^·K^−1^
L	2258	kJ·kg^−1^

**Table 3:** Structural properties of MIL-101 (Cr).

	Specific Surface Area/(m^2^·g^−1^)		Pore Volume/(cm^3^·g^−1^)
	BET	Langmuir	
MIL-101(Cr)	3054	4882	1.734

**Table 4:** Values of SCP and COP.

Evaporation Temperature (°C)	Adsorption Quantity (g·g^−1^)	Desorption Quantity (g·g^−1^)	Cycle Adsorption Quantity (g·g^−1^)	SCP (W·kg^−1^)	COP
15	0.45	0.31	0.14	42.7	0.112
20	0.55	0.4	0.15	43.4	0.144

## Nomenclature

A	Clausius–Claperon equation constant
C_a_	specific heat capacity of the adsorbent, kJ·kg^−1^·K^−1^
*C*_lc_	specific heat capacity of water, kJ·kg^−1^·K^−1^
C_m_	specific heat capacity of stainless steel, kJ·kg^−1^·K^−1^
h_d_	desorption heat, kJ·kg^−1^·K^−1^
L	latent heat of vaporization of water, kJ·kg^−1^
M*_a_*	adsorbent mass, kg
M_m_	adsorption bed mass, kg
Q_c_	heat taken away during cooling of adsorption bed, kJ·kg^−1^
Q_eva_	sensible heat of liquid refrigerant from T_c_ to evaporation temperature T_e_, kJ·kg^−1^
Q_g_	heat absorbed during the desorption process, kJ·kg^−1^
Q_ref_	cooling capacity, kJ·kg^−1^
R	universal gas constant, J·mol^−1^·K^−1^
T_a1_	initial adsorption temperature, K
T_a2_	adsorption temperature, K
T*_c_*	condensation temperature, K
T_e_	evaporation temperature, K
T_g1_	initial desorption temperature, K
T_g2_	desorption temperature, K
T_sat_	saturation temperature corresponding to the adsorption pressure, K
x_conc_	water adsorption capacity in gas–solid-phase equilibrium at the time of adsorption in adsorption temperature, kg·kg^−1^
x_dil_	water adsorption capacity in gas–solid-phase equilibrium at the time of desorption in desorption temperature, kg·kg^−1^
x_0_	maximum adsorption capacity, kg·kg^−1^
Δx	circulating adsorption capacity, kg·kg^−1^
